# Ras and Rab Interactor 3: From Cellular Mechanisms to Human Diseases

**DOI:** 10.3389/fcell.2022.824961

**Published:** 2022-03-14

**Authors:** Ruinan Shen, Caitlin J Murphy, Xiaowen Xu, Mingzheng Hu, Jianqing Ding, Chengbiao Wu

**Affiliations:** ^1^ Institute of Neurology, Ruijing Hospital, Shanghai JiaoTong University School of Medicine, Shanghai, China; ^2^ Department of Neurosciences, University of California San Diego School of Medicine, La Jolla, CA, United States

**Keywords:** RIN3, Rab5, endocytosis, trafficking, Alzheimer’s disease

## Abstract

Ras and Rab interactor 3 (RIN3) functions as a Guanine nucleotide Exchange Factor (GEF) for some members of the Rab family of small GTPase. By promoting the activation of Rab5, RIN3 plays an important role in regulating endocytosis and endocytic trafficking. In addition, RIN3 activates Ras, another small GTPase, that controls multiple signaling pathways to regulate cellular function. Increasing evidence suggests that dysregulation of RIN3 activity may contribute to the pathogenesis of several disease conditions ranging from Paget’s Disease of the Bone (PDB), Alzheimer’s Disease (AD), Chronic Obstructive Pulmonary Disease (COPD) and to obesity. Recent genome-wide association studies (GWAS) identified variants in the *RIN3* gene to be linked with these disease conditions. Interestingly, some variants appear to be missense mutations in the functional domains of the RIN3 protein while most variants are located in the noncoding regions of the *RIN3* gene, potentially altering its gene expression. However, neither the protein structure of RIN3 nor its exact function(s) (except for its GEF activity) has been fully defined. Furthermore, how the polymorphisms/variants contribute to disease pathogenesis remain to be understood. Herein, we examine, and review published studies in an attempt to provide a better understanding of the physiological function of RIN3; More importantly, we construct a framework linking the polymorphisms/variants of RIN3 to altered cell signaling and endocytic traffic, and to potential disease mechanism(s).

## The Ras and Rab Interactor Protein (RIN) Family

### RIN Proteins as the Guanine Nucleotide Exchange Factor for Rab5

**FIGURE 1:**
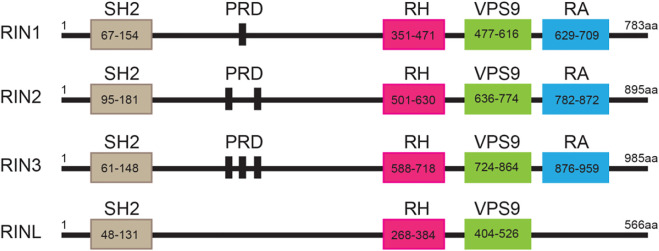
Structure of the RIN protein family. Domain structures of individual RIN proteins. Functional domains with indicated residues are presented by frames. SH2, src homology 2; PRD: proline-rich domain; RH: Rin homology; VPS9: vacuolar protein sorting-associated protein 9; RA: Ras-association. The amino acid residues for each of these domains are marked.

The highly conserved RIN family that includes RIN1, RIN2, RIN3 and the RIN-like protein (RINL). All the RIN members (except RINL) are composed of five functional domains: a Src homology 2 (SH2) domain, a proline-rich domain (PRD), a RIN-homology (RH) domain, a VPS9 domain (vacuolar protein sorting-associated protein 9) and a Ras-association (RA) domain (Figure 1). The only difference among the RIN members lies in the number of PRDs contained; RIN1 has one, RIN2 has two, RIN3 has three, while RINL has none (Figure 1). In 1991, Colicelli and colleagues used a cDNA library to screen for possible interactor(s) that interfered with RAS function in *Saccharomyces cerevisiae* (Colicelli et al., 1991). They discovered that, although RIN1 and two other proteins bound to Ras, they did not suppress the activated form of Ras (Colicelli et al., 1991). Then in 1997, Rab Guanine Nucleotide Exchange Factor 1 (RabGEF1, also called Rabex5) and its yeast homolog Vps21p were identified by their preferential binding to Rab5 in its nucleotide-free state and by catalyzing the GDP/GTP exchange of Rab5 (Bucci et al., 1992; Hama et al., 1999). The conversion of GDP-bound to GTP-bound form results in the activation of Rab5 that promotes endocytosis and endocytic traffic (Bucci et al., 1992; Xu et al., 2016). Thus, Rabex5 was the first guanine nucleotide exchange factor (GEF) identified for the small GTPase Rab5 (Horiuchi et al., 1997). A detailed structural analysis of Rabex5 showed that it contained a Vps9 catalytic domain that was responsible for its GEF activity towards Rab5 (Hama et al., 1999). It was since suggested that any proteins that contained the same modularity and biochemical characteristic of the Vps9 domain may also function as a GEF to activate Rab5 (Hama et al., 1999). Based on these principles, RIN1 (Tall et al., 2001), RIN2 (Saito et al., 2002), RIN3 (Kajiho et al., 2003b; Kajiho et al., 2011) and RINL (Woller et al., 2011; Kajiho et al., 2012), were all identified and confirmed to function as GEFs to promote the activation of Rab5.

### Expression Profiles

**FIGURE 2:**
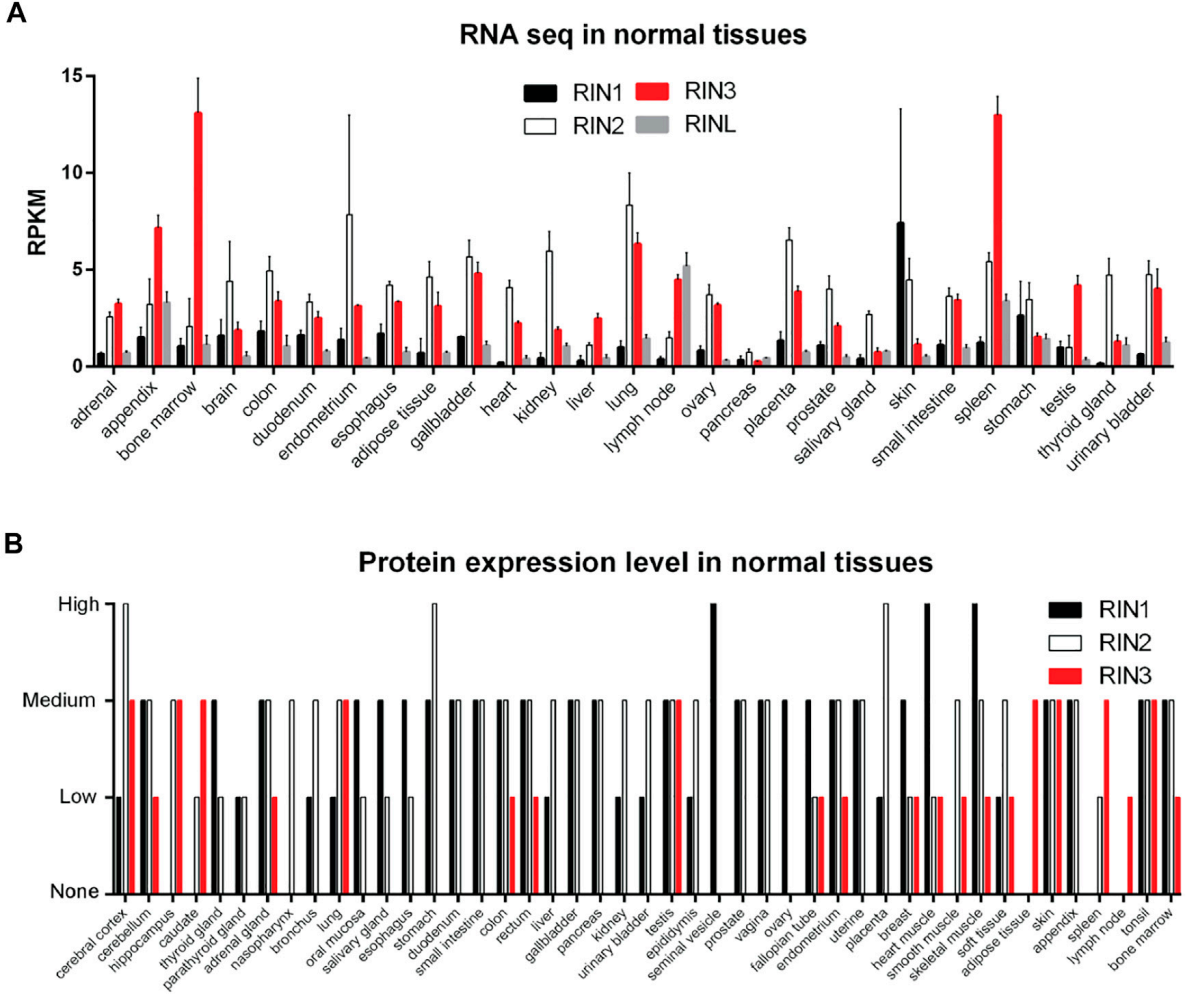
Expression profile of RIN family in normal human tissues. **(A)**: RNA levels of individual RIN genes. Data comes from HPA RNA-seq normal tissues project (Fagerberg et al., 2014). **(B)**: Protein expression level of RIN1/2/3. Data comes from the Human Protein Atlas (https://www.proteinatlas.org/).

All three human RIN mRNA and proteins are widely expressed in human tissues (Figures 2A,B). However, the tissue distribution of RIN3 protein appears to be more selective than RIN1 and 2 (Janson et al., 2012) (Figure 2B). For the purpose of this review, we are mostly focused on RIN3. Human *Rin3* gene is located on chromosome 14q32 and encodes a protein of 985 residues (Figure 1). The RIN3 mRNA is found at the highest levels in the hematopoietic system that includes bone marrow and lymphatic tissues (Uhlén et al., 2015) (Figure 2A). Relatively high expression of RIN3 mRNA is also seen in the nervous systems, the pulmonary and the male reproduction system (Figure 2A). In human tissues, RIN3 protein has been found to be mostly expressed in the peripheral blood cells and also in the brain, muscle, colon and mast cells (Kajiho et al., 2003b; Uhlén et al., 2015) (Figure 2B).

### Domain Structure

#### The Role of VPS9 Domain

The Vps9 domain in human RIN3 (residues 724–864; Figure 1) is found in 18 other human proteins including Rabex5, other members of the RIN family and ALS2 (Amyotrophic Lateral Sclerosis Type 2) and in yeast Vps9p (Hama et al., 1999; Esters et al., 2001; Otomo et al., 2003). Anna Delprato and colleagues constructed a panel of 37 mammalian Rab GTPases and found that the helical bundle-VPS9 domain in Rabex5 showed activity in catalyzing the release of GDP from these Rab proteins. In particular, the Rabex5 VPS9 domain exhibited a potent GEF activity specific for Rab5, Rab21 and weak activity for Rab22, while showing no detectable activity for other Rab proteins (Delprato et al., 2004). Due to the presence of a VPS9 domain in RIN1, it was not surprising that RIN1 was shown to have strong GEF activity for Rab5A, Rab5B, Rab5C and Vps21p (Tall et al., 2001). Kajiho and colleagues employed elegant biochemistry studies and demonstrated that RIN3 also promoted GTP binding of Rab31, another member of the Rab5 family (Kajiho et al., 2003b; Kajiho et al., 2011).

The VPS9 domain in the RIN family proteins is essential for their Rab5 GEF activity, as its deletion in RIN2 completely abolished its capability to bind Rab5 and to catalyze release of GDP (Saito et al., 2002). Furthermore, mutations of four residues (Asp 313, Pro 317, Tyr 354, and Thr 357) in the VPS9 domain of Rabex5 severely impaired its GEF activity for both Rab5 and Rab21 (Delprato et al., 2004). Similarly, double mutations in the respective VPS9 domain of RIN1 (D537A + P541A or Y577A + T580A), RIN2 (D696A + P700A or Y736A + T739A), RIN3 (D785A + P789A or Y825A + T828A) each led to significant reductions in their GEF activity. The Y825A + T828A mutations in RIN3 were especially effective for inhibiting its GEF activity (Kajiho et al., 2011). These findings establish the essential GEF function of VPS9 to activate Rab proteins.

#### The Role of the RH Domain

Although the VPS9 domain is necessary for RIN proteins to interact with and catalyze activation of Rab5, it is not sufficient to accomplish the task; the RH domain (residues 588–718 in RIN3) is also required for RIN3 interaction with Rab5 proteins. Using a yeast two hybrid system to study the impact of different domains of RIN2 on activation of Rab5, Saito et al., demonstrated that deletion of either the RH or the VPS9 domain resulted in a complete loss of interaction between Rab5 and RIN2 (Saito et al., 2002). This is further supported by a previous structural analysis showing that the Rabex5 VPS9 domain appeared to interact with its upstream helical bundle structure (∼88 amino acids) (Delprato et al., 2004; Delprato and Lambright, 2007). Therefore, it is possible that a collaborative effect between the RH and VPS9 domains is necessary in recruiting and catalyzing activation of Rab5 proteins. In addition, when eight serine residues (523, 524, 530, 533, 535, 537, 583, 586) of the internal sequence between SH2 and RH domains were mutated to alanine, RIN3 interaction with Rab31, but not Rab5, was abolished. These residues are theoretically located between the PRDs and closely upstream to the RH domain. It is thus likely that the RH domain may provide specificity in substrate binding to VPS9 (Kajiho et al., 2011). Functionally speaking, it is possible that the RH region is required for interacting with Rab5 family members and the VPS9 domain is critical for its GEF activity of RIN proteins, thus deletion of either domain will abolish RIN GEF activity.

Despite all RIN family proteins are believed to catalyze the GDP/GTP exchange for the Rab5 family proteins (including Rab5A/B/C, Rab21, Rab 22, and Rab31), each member may show slight preference for different Rab5 family members. For example, RIN3 had relatively higher GEF activity towards Rab31 when compared to RIN1, 2 (Kajiho et al., 2011). In addition, some interesting differences also exist among the members of the Rab5 family that may reflect the heterogenous nature of the early endosomal population; and each Rab5 family member may reside within a subpopulation of early endosomes. Because of its strong GEF activity towards Rab5, expression of exogenous RIN3 induced enlarged early endosomes, mediated by the early endosome antigen 1 (EEA1) (Simonsen et al., 1998). However, RIN3 showed a greater degree of colocalization on early endosomes with Rab5, than with EEA1 (Kajiho et al., 2003b). Interestingly, overexpressed RIN3 was found to be colocalized with Rab31, and RIN3/Rab31 exhibited partial overlap with EEA1, suggesting that RIN3/Rab31 were partially localized on EEA1-positive early endosomes (Kajiho et al., 2011). Furthermore, following internalization, transferrin was observed to stay within the RIN3/Rab5-positive vesicles for 4–10 min. By 15 min, the transferrin was found in the EEA1-positive vesicles; yet the transferrin receptor was still partially contained within the RIN3- and Rab31-positive vesicles (Kajiho et al., 2003a; Kajiho et al., 2003b; Kajiho, 2003). These results indicated that RIN3 may participate specifically in the transport steps between the cell membrane and EEA1-positive early endosomes (Kajiho et al., 2003b). More detailed studies will be needed to determine and differentiate the different roles of RIN3 in endocytosis and endocytic traffic (Kajiho et al., 2011).

#### The RA Domain

RIN proteins all contain a Ras association domain (RA) even though these sequences are not exactly the identical among the RIN members. RIN1 was first observed as an interactor for activated Ras in yeast (Colicelli et al., 1991) and was later shown to compete with Raf1 to bind Ras protein *in vitro* (Han and Colicelli, 1995). Overexpression of the RIN1-RA domain in mammalian cells blocked Ras mediated signaling (Han et al., 1997). Intriguingly, RIN1 has a natural truncated splicing form which is missing the 429–490 amino acid residues (right at the N terminal of the VPS9 domain). This truncated RIN1 lacked Rab5 GEF activity and showed weaker binding to Ras and 14-3-3 protein as well (Han et al., 1997), suggesting that these 61 amino acid residues of RIN1 were somehow required for the RA-mediated Ras interaction as well. Unlike RIN1, RIN2 and RIN3 have been rarely studied with respect to their role in Ras function and signaling. Nevertheless, because of their high sequence homology, the RA domains in RIN2/RIN3 may have similar functions.

Unfortunately, there has been no study to determine the relationship between Ras activation and RIN3 activity as of to date. Evidence for their interaction or how Ras activation induces RIN3-mediated endocytic alternation is still missing. Hypothetically, RIN3 might bind Ras *via* RA domain, and activated Ras might also promote Rab5 activation to mediate receptor internalization and endocytic trafficking, which makes RIN3 another bridge for Ras-cell signaling and Rab5-endocytosis process.

Unfortunately, there is no research ever tried to determine the relationship between Ras and RIN3 to date. Evidence for their interaction or Ras induced RIN3 mediated endocytic alternation are still missing. Hypothetically, RIN3 might bind Ras via RA domain, and activated Ras might also promote Rab5 activation and endocytic receptor mediated internalization, which makes RIN3 another bridge for Ras-cell signaling and Rab5-endocytosis process (Tall et al., 2001).

#### The SH2 Domain

The SH2 domain plays a key role in organizing the cellular signaling network by protein tyrosine kinases (PTKs) (Pawson et al., 2001; Schlessinger and Lemmon, 2003). RIN1 itself contains several tyrosine residues that are phosphorylated by the c-ABL tyrosine kinase (Han et al., 1997). The SH2 domain of RIN1 interacts with the ABL2 non-receptor tyrosine kinase, through which RIN1 stimulates the ABL2 kinase activity to facilitate the Ras-RIN1-ABL2 signaling pathway (Hu et al., 2005). Under unstimulated conditions, RIN1-SH2 binds to the epidermal growth factor receptor (EGFR) and inhibited EGF uptake; Upon EGF stimulation, activated EGFR promoted the formation of the Ras-RIN1-Rab5 complex both on the plasma membrane and in the cytoplasm. Recruitment of this complex to the plasma membrane may subsequently initiate early stage of the endocytosis (Barbieri et al., 2003).

The Rab5 GEF activity of RIN3 is likely modulated by tyrosine phosphorylation, as treatment of Hela cells with pervanadate (PV), a non-selective tyrosine-phosphatase inhibitor, induced the translocation of RIN3 from the cytoplasm to the Rab5-positive vesicles (Yoshikawa et al., 2008). Although the tyrosine phosphorylation site(s) of RIN3 was not determined (Yoshikawa et al., 2008), these data provide evidence that the RIN3 GEF activity is modulated by tyrosine kinase signaling, through which RIN3 might bridge the cellular signaling transduction events with the endocytic process.

**FIGURE 3:**
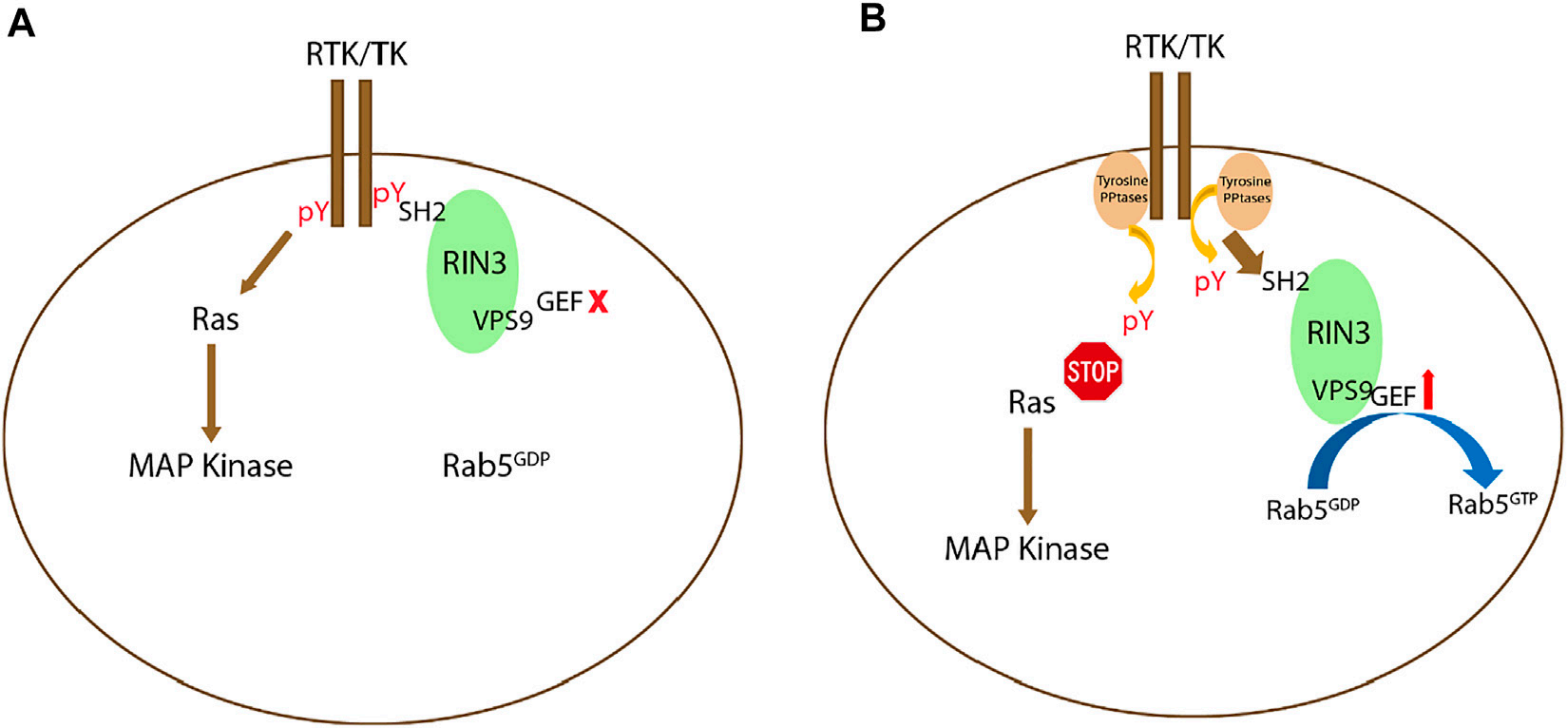
Potential signaling mechanism(s) regulating RIN3 GEF activity. **(A)**: Upon activation of receptor tyrosine kinase (RTK) or tyrosine kinase (TK), while the Ras/MAP kinase signaling cascade is activated, RIN3 also via its SH2 domain binds to the phosphor-tyrosine residue (pY) leading to suppression of the GEF activity of RIN3 and Rab5 stays as GDP bound. **(B)**: Deactivation of RTK/TK by the phosphotyrosine phosphatase (PPtase) results in attenuation of the Ras/MAP kinase signaling. Concomitantly, RIN3 is decoupled and breaks free from RTK/TK, which in turn activates the GEF activity to promote the conversion of Rab5 from GDP- to GTP-bound form.

Deletion of the SH2 domain in each of the three RIN proteins was used to evaluate its function. GEF activity increased after SH2 deletion in RIN1 and RIN2 compared to their full-length form (Tall et al., 2001; Saito et al., 2002). RIN3 had reduced colocalization with Rab5 after SH2 deletion (Yoshikawa et al., 2008). All these results point to a mechanism by which the SH2 domain may self-inhibit the GEF activity of RIN proteins. It is possible that at the onset of activation, receptor tyrosine kinases (RTKs) bind to the RIN SH2 domain(s) to suppress their GEF activity and to facilitate signal transduction; inactivation of RTKs by tyrosine phosphatase(s) then results in decoupling RTKs from RIN SH2. As such, the GEF activity is disinhibited to initiate endocytosis and endocytic traffic. Further efforts are needed to test this hypothesis (Figure 3).

#### The Proline-Rich Domains

The number of PRDs is the most obvious difference between the RIN proteins (one in RIN1, two in RIN2 and three in RIN3) (Figure 1). PRD is a functionally important motif that can bind to multiple proteins via SH2 and SH3 domains. The “PxPxPR” sequence in two of the three RIN3 PRDs was identified to be the key structure that binds to two SH3 domains of the CD2-assosiated protein (CD2AP) (Rouka et al., 2015). Similarly, the SH3 domain of bridging integrator 1, BIN1 (also known as amphiphysin I) interacted with the N-terminal fragment (amino acids 1–586) of RIN3, that also encompassed the three PRDs (Kajiho et al., 2003b). It is thus not surprising that RIN3 recruited CD2AP and BIN1 to Rab5 positive vesicles (Shen et al., 2020), but the impact of this interaction on the RIN3 GEF activity and the cellular function of this complex is unknown. In addition, BIN2 was found to interact with RIN3 in the LAD2 human mast cell line (Janson et al., 2012). Given that BIN1 and BIN2 share both the analogous Bin1/amphiphysin/Rvs167 (BAR) and SH3 domain (Ren et al., 2006), it is likely that BIN2 also binds to the RIN3-PRD via its SH3 domain. The interaction of RIN3 and BIN2 was diminished under stem cell factor (SCF) stimulation but can be restored after recovery from the stimulation for 4 h (Janson et al., 2012). These results suggests that interaction between RIN3 and BIN1/BIN2 can also be regulated by RTKs signaling.

#### Potential Self-Interaction

The PRDs (residues 207–496) of RIN2 also interact with full length RIN2, while the VPS9-PRD domain (residues 642–895) also showed positive but weak interaction (Saito et al., 2002). Both the N-terminal SH2 domain and the C-terminal RA domain of RIN2 displayed strong interaction with themselves, which suggest that two RIN2 molecules might form parallel complexes in a head-to-head and tail-to-tail configuration. Both the PRDs and the N-terminal region (207–297 amino acid residues between the SH2 and PRDs) were required to bind the C-terminal RH, VPS9 and RA domains. Thus, RIN2 might also form antiparallel complexes in a head-to-tail configuration. Alternately RIN2 can fold itself to accomplish self-interaction between its N- and C-terminal region (Saito et al., 2002). Given the sequence similarity among the RIN proteins, it is likely that RIN1 as well as RIN3 each possess some self-interaction ability. Although RIN3 dimerization, either in cell lines or in mouse brain samples, was not detected in a recent study (Shen et al., 2020), further structural analysis, biochemistry and cell biology studies are needed to fully clarify all these hypotheses and to define the function of these interactions.

## RIN3 and Human Diseases

RIN3 has been linked to multiple human diseases such as Paget’s Disease of bone (PDB), Alzheimer’s Disease (AD), Chronic Obstructive Pulmonary Disease (COPD), obesity, sickle cell disease etc. It is perhaps not surprising that all these diseases involve tissues or organs that have high expression of RIN3.

### RIN3 and Paget’s Disease of bone

Paget’s Disease of bone (PDB) is the second most common metabolic bone disorder after osteoporosis. PDB patients suffer from a variety of symptoms ranging from softer bones to increased bone growth; The enlarged bone growth typically involves one or more bones of the pelvis, low back (spine), hips, thighs, head (skull) and arms. Although most patients are asymptomatic throughout the disease evolution, pain, bone deformity, pathological fracture and deafness are common clinical manifestations (Tan and Ralston, 2014). Increased bone turnover induces larger bone volume, such that patients often complain about enlarged feet or head because of abnormal bone thickening (Seitz et al., 2009). Osteoclasts are increased in both size and number, that may elevate osteolytic activity, leading to enhancement of bone formation (Roodman and Windle, 2005). Presently, there is no effective treatment for PDB except for pain control in clinic practice. The disease etiology and pathogenesis remains largely undefined (Alonso et al., 2017).

Genetic factors play an important role in PDB. 7–30% patients have positive family history with an autosomal dominant mode of transmission (Morales-Piga et al., 1995; Hocking et al., 2000; Hocking et al., 2001; Laurin et al., 2001). Mutations in Sequestosome 1 (SQSTM1)/p62 protein, a ubiquitin signaling and autophagy receptor, were first identified as a genetic cause of PDB (Hocking et al., 2002; Laurin et al., 2002).

Recent genome-wide associated studies (GWAS) have identified seven loci with robust association with PDB (Albagha et al., 2010; Albagha et al., 2011). Among them, rs10498635 C allele on chromosome 14q32.12 was strongly associated with PDB in several European populations (*p*-value = 2.55 × 10^–11^, odds ratio OD = 1.44 (1.29–1.60). The region contains only the RIN3 gene and 62kb upstream of the *RIN3* coding region. Through targeted sequencing of the 60kb region in the *RIN3* gene in samples from 741 PDB patients and 2,699 healthy controls, Vallet et al. (Vallet et al., 2015) identified 18 PDB-associated variants in *RIN3* including 16 missense variants. Among the 16 missense variants, 9 loci located in the PRDs, 1 in the SH2 domain and 3 in the VPS9 domain (Vallet et al., 2015). Three missense variants are most common in PTB: the rs117068593 T allele, the rs117068593 C allele and the rs10498635 C allele. The rs117068593 T allele was carried by more individuals in the healthy control group than in the PDB group (OR = 0.68; 95% CI = 0.58–0.81; imputed *p* = 5.7 × 10^–6^). The rs117068593 C allele and the rs10498635 C allele both displayed strong linkage disequilibrium, indicating that these two variants may play a pathogenic role in PDB (Vallet et al., 2015). Many of the rare variants identified in this study were more common in the PDB cases than with the healthy controls. Although these variants did not reach statistical significance individually, the results were highly significant when they were combined (OR = 3.72; 95% CI = 2.38–5.82; *p* = 8.9 × 10^–10^) (Vallet et al., 2015).

**FIGURE 4:**
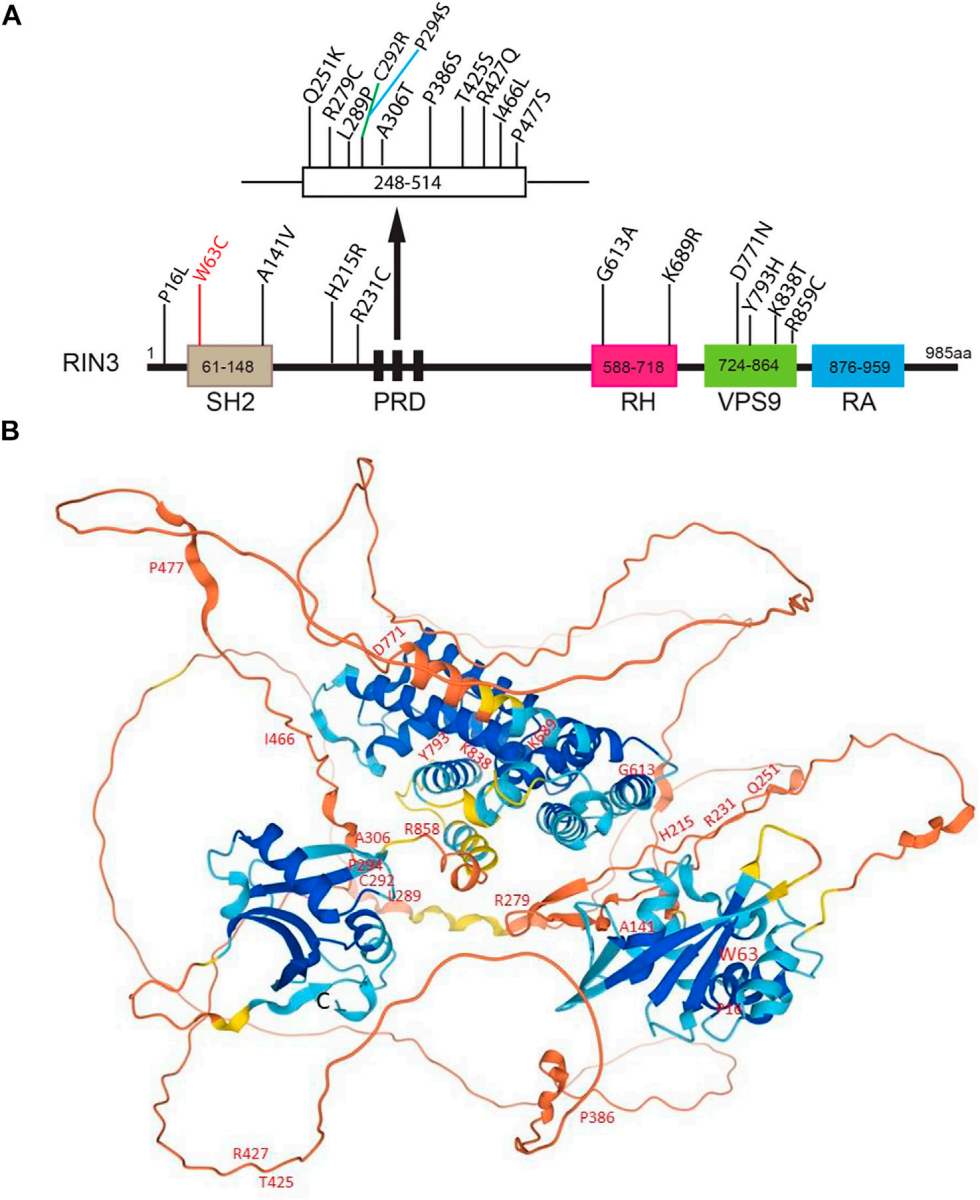
Disease-associated variants/mutations and predicted structure of human RIN3. **(A)**. A linear presentation of all known mutation sites in the various domains of RIN3. Missense mutations in RIN3 have been discovered thus far, all are associated with Page’s Disease of the Bone; except that: 1) W63C is linked with sporadic EOAD; 2) R279C is associated with COPD and P386S. **(B)**. Predicted structure of human RIN3 by AlphaFold (https://alphafold.ebi.ac.uk/entry/Q8TB24). All the known mutations that have been identified are marked. For a detailed prediction of RIN3 3D structure, see supplemental movie.

22 distinct variants were recently identified in a Belgian population. Eight of them were newly recognized missense variants, and two were in the UTRs (De Ridder et al., 2019). The N terminal variant P16L was found only in one control individual. The W63C variant in the SH2 domain was found in two control individuals but not in any PDB patients, despite the fact this variant was predicted to be deleterious by all predictive tools. Interestingly, W63C was also identified to be a risk factor for sporadic early onset Alzheimer’s disease (sEOAD) (Kunkle et al., 2017). A series of rare variants (p.K689R, p.Y793H, p.K838T and p.R859C) were exclusively found in patients and were all located in the RH and the VPS9 domain (Figure 4A). The P386S variant probably diminishes interaction between the PRDs and the SH3 domain (Rouka et al., 2015). Mutations in the SH2 domain (W63C and A141V) do not seem to disrupt interaction between the PRDs and the SH3 domain (Figure 4A).

Presently, the function of RIN3 in bone biology and homeostasis remains unknown. However, an intron variant rs754388 was found to associate with lower and upper limb bone mineral density (BMD), and this variant was in high linkage disequilibrium with common PDB variant rs117068593. This suggests a significant role for RIN3 in the regulation of bone mass development and maintenance of skeletal health (Kemp et al., 2014). Similar association between rs754388 and bone mineral density (BMD), a heritable, polygenic trait, was identified (Medina-Gomez et al., 2017).

As discussed earlier, RIN3, as a Rab5 GEF, regulates Rab5 GTPase to impact intracellular transport pathways. Rab GTPases have been strongly implicated in the regulation of osteoclast function and bone resorption (Itzstein et al., 2011; Weivoda and Oursler, 2014). For instance, a.

Mouse model that harbors a mutation in the catalytic subunit of the Rab geranylgeranyl transferase (RGGT) showed a loss of 75% of the activity of this enzyme and hence hypoprenylation of several Rabs (Rab2B, Rab3D, Rab5, Rab6, Rab7, and Rab14) in osteoclasts. Prenylation is required for these Rab proteins to associated with membranes; hypoprenylation may thus reduce membrane attachment and decrease the ability of Rabs, affecting intracellular transport pathways (Alexandrov et al., 1994; Goody et al., 2005). Although osteoclasts from the RGCT mice showed normal differentiation and polarization, they exhibited reduced resorptive activity *in vitro* (Taylor et al., 2011). Consistently, disruption of Rab geranylgeranylation with a phosphonocarboxylate inhibitor of RGGT inhibited osteoclast bone resorption (Coxon et al., 2001). Thus, deleterious missense mutations found in RIN3 may disrupt Rab5 activity, that could result in osteoclast dysfunction.

Using a mouse model with a targeted inactivation of the mouse Rin3 gene, Vallet et al. (Vallet et al., 2021) showed that the homozygous RIN3 knock out (−/−) mice had normal weight compared to their wild type controls. However, micro-computer tomography (microCT) analysis revealed that, comparing to the wildtype control, the RIN3^−/−^ mice showed a significant increase in bone mass and trabecular number, but a significant decrease in trabecular separation (Vallet et al., 2021). Although the number of osteoclasts in the trabecular bone remained unchanged in bone sections, the surface occupied by Tartrate-resistant acid phosphatase staining (TRAcP)-positive osteoclasts was significantly reduced in the RIN3^−/−^ mice (Vallet et al., 2021). These results suggest that the RIN3^−/−^ mice may have fewer active osteoclasts. It is thus likely that RIN3 deletion interferes with osteoclast activity and reduce bone resorption, thus leading to increased bone mass.

In summary, multiple mutations in RIN3 have been identified to associate with elevated risk of PDB. However, whether these mutations function as gain or loss of function to contribute to the pathogenesis of PDB remains unclear. Although some domains whose structures have been predicted with high precision by AlphaFold (Jumper et al., 2021; Varadi et al., 2021) (Figure 4B), also see Supplementary Movie S1), the 3D structure of RIN3 is far from being resolved. How the mutation(s) alters the structure and function of RIN3 in PDB and other conditions as discussed below will be hopefully answered in the future.

### RIN3 and Alzheimer’s Disease

Alzheimer’s Disease (AD) is the most common progressive neurodegenerative disorder whose clinical manifestations included late memory loss and cognitive impairment (Selkoe and Hardy, 2016). The classical neuropathological hallmarks for AD include Aβ-amyloid-containing neuritic plaques and phosphorylated Tau-containing neurofibrillary tangles (NFT) (Karch et al., 2014). In addition, significant synaptic loss, selective neuronal death, neurotransmitter defects and neuroinflammation are also observed in AD (Karch and Goate, 2015; Barragán Martínez et al., 2019). Despite the tremendous efforts and investments in the past decades, there are currently no disease-modifying therapy for effective treatments of AD (Doody et al., 2013a; Doody et al., 2013b; Gauthier et al., 2016).

As a multifactorial disease, genetic background proves to play an important role in AD pathogenesis. In 2009, apolipoprotein E (*APOE*), which functions in lipid transport, Aβ traffic, synaptic function, immune regulation, and intracellular signaling (Mahley et al., 2008), was identified as a risk factor for late onset AD (LOAD) (Gatz et al., 2006). More recently, large scale GWAS and a meta-analysis of LOAD patients have identified more than 20 additional risk factors (Harold et al., 2009; Seshadri et al., 2010; Sherva et al., 2011; Chapuis et al., 2013). The identification of these additional risk factors further illustrates the extraordinary genetic complexity of AD.

A significant number of the newly identified loci (PICALM, BIN1, EPHA1, CD2AP, MEF2C, PTK2B, SORL1 and RIN3) encode protein products with significant roles in endocytic traffic and signaling (Karch and Goate, 2015). The involvement of RIN3 in LOAD was revealed by the identification of a locus (rs10498633, G/T), upstream of the coding sequences within the enhancer region of the *Rin3* gene, but downstream of the SLAC24A4 gene (Chapuis et al., 2013; Lambert et al., 2013). This single nucleotide polymorphism (SNP), also known as the SLC24A4/RIN3 SNP, was speculated to cause an increase in the expression of RIN3 in LOAD patients.

When comparing extreme AD cases with centenarian controls, the variant effect size for the SLC24A4/RIN3 SNP increased by 4.5-fold, versus 2-fold for APOE-ε4 and 6.5-fold for TREM2 (R74H) variant in comparison to published effect sizes (Tesi et al., 2019). Stage et al. (2016) used elegant brain imaging to analyze the effect of the top 20 AD risk genes on gray-matter density and FDG PET brain metabolism By analyzing a cohort of 381 normal controls, 634 mild cognitive impairment (MCI) patients and 243 dementia patients, they determined that the RIN3 risk variant rs10498633 G/T was significantly associated with mean median temporal lobe grey matter density (GMD) (Stage et al., 2016). The RIN3 variant was also a significant predictor of hypometabolism in normal control group (Stage et al., 2016).

In a recent study, *PICALM* (rs10792832), *BIN1* (rs6733839), *CD2AP* (rs10948363), and *RIN3* (rs10498633) were sequenced in a Danish cohort of 74,754 individuals from the general population (Juul Rasmussen et al., 2019). The results were used to generate a weighted and a simple allele score. The analysis has revealed that all four variants were associated with increased risk of AD and all other dementia cases including vascular dementia. Furthermore, the effect of these risk factors was independent of the strong *APOE*-ε4 allele (Juul Rasmussen et al., 2019).

A more recent case-control study of whole-exome sequencing of 93 patients with sporadic early onset AD (sEOAD) has discovered a missense *Rin3* variant (*rs150221413*, G/T). The mutation results in a substitution of 63W > C in the SH2 domain of RIN3 (Kunkle et al., 2017). The RIN3^W63C^ variant is strongly associated with sEOAD (OR, 4.56; 95% CI, 1.26–16.48; *p* = 0.02, BP = 0.091) (Kunkle et al., 2017). In an analysis of 624 Taiwanese people with an average age of 64.2 years, Lin and colleagues identified 11 *Rin3* variants that were markedly correlated with the Mini-Mental State Examination (MMSE) score (*p* < 0.05) (Lin et al., 2017). Among them, an intron-variant *rs1885747* on *Rin3* was found to be positively correlated with the MMSE score in the cohort (Lin et al., 2017), although it is not clear the effect of this variant on the expression of RIN3 presently.

The potential involvement of RIN3 in AD pathogenesis is also supported by epigenetic studies. In a 2017 study of gene methylation profiling of blood and brain samples from 22 AD and 26 normal control subjects (27 males, 21 females), the AD samples showed significant group-wide hypomethylation of 7 CpGs located within the 3′UTR region of *Rin3* (CpG1 *p* = 0.019, CpG2 *p* = 0.018, CpG3 *p* = 0.012, CpG4 *p* = 0.009, CpG5 *p* = 0.002, CpG6 *p* = 0.018, and CpG7 *p* = 0.013, respectively) (Boden et al., 2017). The effect was specific for *Rin3* since other LOAD risk factors (PTK2β, ABCA7, SIRT1, or MEF2C) did not show significant changes in methylation of their respective promoters. Additionally, a genome wide methylation study of Mexican Americans with mild cognitive impairment uncovered significant hypo-methylation in RIN3 and three other genes (Pathak et al., 2019). Together, these studies have suggested the possibility that increased expression of wildtype RIN3 or expression of the RIN3 variant (W63C) may contribute to AD pathogenesis. However, the underlying mechanism(s) is presently unknown.

**FIGURE 5:**
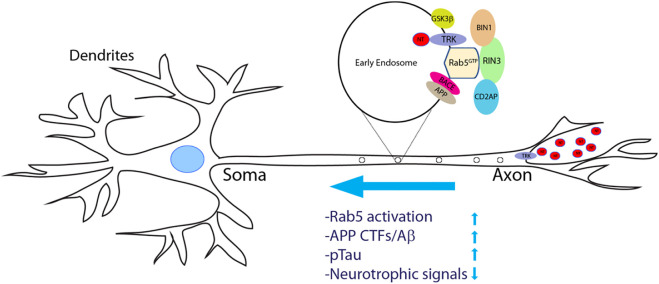
A proposed model of increased RIN3 activity in disrupting axonal transport in Alzheimer’s Diseases. Neurotrophic factors (NTs) bind to and activate their respective tyrosine receptor kinases (TRKs) to form a signal complex, that is internalized into Rab5+ early endosomes for retrograde transport. Increased expression of RIN3 or the W63C variant enhances Rab5 activation, which in turn promotes APP processing to produce c-terminal fragments (CTFs)/Aβ in conjunction withCD2AP. In addition, RIN3/BIN1 increases phosphorylation of Tau. Consequently, retrograde transport of trophic signals (NT/Trk) is impaired leading to eventual neurodegeneration in AD.

An important question is how dysregulation of RIN3 expression and function contributes to AD pathogenesis. Given that neurons have the most extraordinary architecture with elaborate dendrites/axons which undoubtedly impose a significant need for endocytic processes, it is not surprising that these early endocytic pathways are found to be dysregulated in AD (Cataldo et al., 2000; Ginsberg et al., 2010; Ginsberg et al., 2011; Nixon, 2005). Persistent hyperactivation of Rab5 was observed in early phases of AD (Ginsberg et al., 2010; Ginsberg et al., 2011; Kim et al., 2016; Xu et al., 2016). Under normal conditions, Rab5-early endosomes are a major site of APP processing by β-secretase (BACE1) to yield the β-cleavage C-terminal fragment (βCTF), which is further processed in late endosomes/trans-Golgi network (TGN) to give rise to Aβ (Cataldo et al., 2004; Kinoshita, 2003; Nixon, 2005; Thinakaran and Koo, 2008; Xu et al., 2016). Therefore, traffic and processing of APP to produce toxic βCTFs/Aβ is intimately regulated by the endocytic pathways (Grbovic et al., 2003). Studies have demonstrated that neurotrophic factors such as nerve growth factor (NGF) and its receptor TrkA are engulfed into early endosomes and are retrogradely transported from the presynaptic terminal to soma (Figure 5). This process is vital for neuronal survival and function (Howe et al., 2001). It is the Rab5 positive vesicles that carry and move the trophic signaling complex in the process (Delcroix et al., 2003; Xu et al., 2016). Thus, dysregulation of Rab5 traffic may deprive neurons of vital survival signals that leads to neurite retrieval and degeneration (Laifenfeld et al., 2007; Xu et al., 2016). Hence, increased expression level or elevated activity of RIN3 might account for the upregulation of Rab5 activation in these studies, potentially leading to axonal transport impairments (Figure 5).

In addition, we have recently demonstrated that RIN3 interacts with the SH3 domains of CD2AP and BIN1, two known LOAD risk factors (Shen et al., 2020). The tripartite RIN3/BIN1/CD2AP complex may play an important role in AD pathogenesis (Shen et al., 2020). Overexpression of RIN3 recruited CD2AP and BIN1 to Rab5 positive early endosomes (Shen et al., 2020). CD2AP, a membrane-associated scaffolding protein, likely controls the assembly of protein complexes and participates in endocytosis and endocytic traffic to transmit intracellular signals (Harrison et al., 2016). CD2AP, by virtue of its multiple protein-protein binding modules, interacts with multiple proteins involved in diverse biological processes (Harrison et al., 2016). A previous study has shown that suppression of CD2AP resulted in a decreased Aβ_42_/Aβ_40_ ratio both in N2a neuron-like cell lines and in 1 month-old APP/PS1 mice (Liao et al., 2015). Additionally, deletion of CD2AP also decreased APP CTFs (Ubelmann et al., 2017). Together, these studies have suggested a role for CD2AP in amyloid pathogenesis.

The longest isoforms of BIN1, expressed predominantly in the central nervous system, contain a unique clathrin-AP2 binding region (CLAP) (Chapuis et al., 2013). Although the role of BIN1 in regulating APP processing is currently under debate (Calafate et al., 2016; Ubelmann et al., 2017; Crotti et al., 2019), BIN1 interacts with Tau (Calafate et al., 2016; De Rossi et al., 2016; Crotti et al., 2019), indicating its potential role in regulating Tau biology. This was confirmed in a recent study, which found that increased BIN1 expression disrupted eye morphology by modulating Tau pathology in *Drosophila* rather than Aβ42 pathology (Kingwell, 2013). Hence, RIN3 may act as a scaffold between CD2AP and BIN1: promoting amyloid pathology via CD2AP and enhancing tau pathology via BIN1.

**FIGURE 6:**
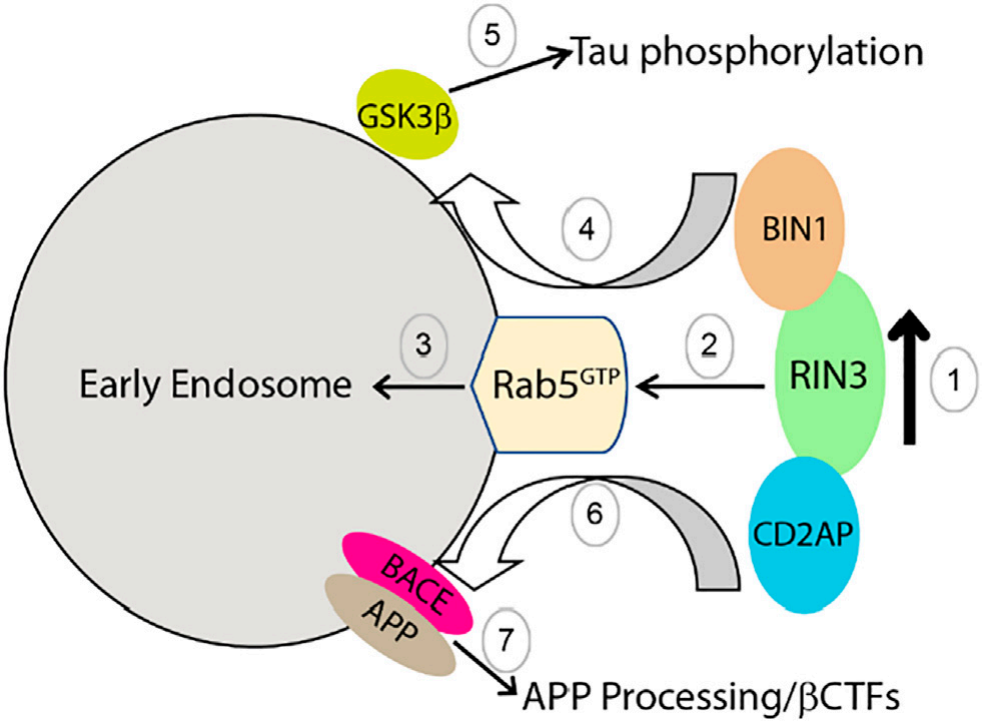
Proposed mechanism by which increased RIN3 activity leads to tau phosphorylation and amyloidic processing of APP in AD.

Based on our current knowledge, we propose the following model to explain how dysregulation and dysfunction of RIN3 contributes to AD pathogenesis by impacting intracellular traffic of Rab5 early endosomes; RIN3 activates Rab5 to promote amyloidogenic processing of APP and tau phosphorylation. Specifically, we postulate that RIN3 forms a complex with BIN1 and CD2AP (Figure 6, step 1). Increased activity of RIN3 enhances Rab5 activation and induces the formation of the RIN3-BIN1-CD2AP complex on early endosomes, that results in impaired endocytic trafficking (Figure 6, step 2,3). Therefore, RIN3-BIN1 stimulates GSK3β to induce tau phosphorylation (Figure 6, step 4, 5); on the other hand, impaired traffic results in processing of APP by BACE1 in early endosomes (Figure 6, step 6, 7).

Currently, the exact role played by the newly identified RIN3 W63C variant in sEOAD is not clear. This particular variant is predicted to be deleterious by multiple prediction tools (De Ridder et al., 2019). Since W63 is located in the beginning of the SH2 domain of RIN3, as discussed earlier, binding of the SH2 domain to phosphorylated tyrosine residues in TKs would suppress the GEF activity of RIN3. It is possible that the W63C mutation weakens the binding of SH2 domain to these phosphotyrosine residue targets and as such, the Rab5 GEF activity in RIN3 is dis-inhibited (Yoshikawa et al., 2008) (Figure 3). The W63C variant, in effect, would result in hyper-activation of Rab5 (Figure 3), which in turn, promotes APP processing and tau phosphorylation (Figure 6). Future studies will be needed to investigate this attractive hypothesis.

### RIN3 and Chronic Obstructive Pulmonary Disease

Chronic Obstructive Pulmonary Disease (COPD) is a common pulmonary disease that is characterized by persistent airflow obstruction. The limitation on airflow is often progressive and associated with abnormal inflammation response of the lung to noxious particles or gases (Fabbri et al., 2003). The onset of the disease normally occurs in midlife and is strongly associated with tobacco smoking history. Patients present with complex pulmonary symptoms such as shortness of breath, wheezing, chronic cough, frequent respiratory infection, etc. Post bronchodilator lung function is used to diagnose and classify COPD. The pathological change in COPD is a mixture of small airway alteration, parenchymal destruction (emphysema), and, in many cases, increased airway responsiveness (asthma) (Fabbri et al., 2003). This disease can be largely preventable and treatable because multiple risk factors are related to smoking and air quality (Mannino and Buist, 2007).

However, patients with similar exposure to tobacco smoking can vary greatly in disease severity and their response to intervention, suggesting that individual genetic diversity may strongly impact the disease progression. Multiple GWAS analyses were performed and RIN3 was identified as a risk factor. In 2014, a genome-wide association case-control study of severe COPD was carried out; the results revealed that *Rin3* rs754388 allele C was a risk allele [*p* = 5.25 × 10–9, OR = 1.28 (1.18–1.39)] (Cho et al., 2014). A meta-analysis then found this variant to have a significant association with pulmonary FEV1/FVC function (Forced Expiratory Volume in 1 s/Forced Vital Capacity) (Lutz et al., 2015). In addition, another missense variant rs117068593 (R279C) was identified to be associated with pulmonary function FEV_1_ (Forced Expiratory Volume in 1 s) (Soler Artigas et al., 2015).

Perhaps it is not surprising that RIN3 mutation is associated with pulmonary disease due to its high expression level in the lung. However, two other RIN3 variants identified in this study are also associated with PDB and BMD (See discussion in RIN3 and Paget’s Disease of Bone), but not with AD. Although very little is known regarding RIN3 function in pulmonary tissue, small GTPases Rab5 and Rab7 are upregulated in alveolar macrophages (Subramaniam et al., 2016). The upregulation of Rab5 and 7 was impaired by second-hand cigarette smoke and this effect can be rescued with recombinant granulocyte–macrophage colony-stimulating factor (GM-CSF). These studies suggest that these Rab proteins may associate to damage repair process in tobacco induced pulmonary conditions (Subramaniam et al., 2016). Dysregulation of RIN3 may thus exacerbate the damages by tobacco in COPD.

### RIN3 and Metabolic Syndrome

Obesity and metabolic syndrome (MetS) are crucial risk factors for cardiovascular disease, diabetes mellitus (DM), hypertension, etc. The heritability of obesity and MetS are respectively 40–70 and ∼30%. Multiple GWAS have been conducted in different human populations and more than 100 loci have been identified. In 2017, an exome-wide association study (EWAS) in the Japanese population identified a new locus on *Rin3*, rs8018360, that was associated with obesity (*p* = 6.79 × 10–9). The rs8018360 can be either T or G, but the T allele was more commonly found in patients, thus defined as a risk allele (Yamada et al., 2017). This variant is located towards the end of the possible promotor region of *Rin3*. Its specific role in expression regulation or RNA splicing is still unknown.

Using next-generation RNA sequencing technology, Padilla and colleagues found that the RIN3 mRNA level in artery tissues was consistently downregulated in obese rats compared to normal rats (Padilla et al., 2014). Although the function of RIN3 in adipose tissue and metabolic pathways is unclear, its downstream Rab5 protein plays a role in these processes. Rab5 activity modulates transport of glucose transporter isoform 4 (GLUT4). Rab5 may also play a role in insulin resistance, a common phenomenon in obesity and MetS (Tessneer et al., 2014). It is thus possible that RIN3 regulates insulin signaling via the Rab5 endocytic pathway.

## Concluding Remarks

RIN3, acts as a GEF for Rab5 to regulate endocytosis and endocytic traffic. By interacting with RAS via its RA domain or with RTKs via its SH2 domain, RIN3 also participates in multiple cellular signaling pathways. Recent evidence has suggested that dysfunction of RIN3 is associated with several human diseases such as Paget’s disease of bone, Alzheimer’s disease, chronic pulmonary obstructive disease and obesity. All disease associated-missense mutations are located in the functional domains and thus could alter the function of RIN3 protein. On the other hand, most *Rin3* polymorphisms and variants are found in the noncoding regions (likely to be promotors or enhancers), which may impact the expression level of RIN3.

A significant issue that needs to be resolved is how the different mutations of RIN3 lead to or contribute to the pathogenesis of very different human diseases. Although we speculate that, through activation of Rab5, increased expression of RIN3 contributes to LOAD and the W63C variant to EOAD, it is far from clear if the same mechanism that also operates for other diseases. Given the role played by RIN3 in endocytic trafficking, it is plausible that specific mutations may alter Ca^2+^ metabolism that causes PDB; whereas in other cases, RIN3 missense mutations/variants affect reabsorption and repair of damaged lung tissues in COPD; Some other mutations may selectively affect cellular insulin signaling and trafficking in DM. Defining the cellular and signaling mechanisms by which the different mutations alter the cellular function of RIN3 will be critical to answer these important questions. Future studies are needed to focus on both defining the structure and delineating cellular interactomes of RIN3 under both physiological and pathological conditions. These efforts will generate insights into its role in disease pathogenesis and inform novel targets for developing effective therapies for these diseases.
